# 

**Published:** 1895-11-23

**Authors:** 


### EARLY EDITION.

**Notices of Births, Deaths, and Marriages** are inserted in this column at a charge of 2s. 6d. for an announcement not exceeding four lines.

**Notice to Advertisers and Subscribers.**

All Advertisements, Orders for Copies of The Hospital and Business Communications should be addressed to **THE MANAGER, (Not to The Editor),** The Hospital, 428, Strand, London, W.C.

The Cost of Subscription, post free, is as follows:—Per week, 2½d.; per quarter, 2s. 8d.; per half-year, 5s. 3d.; per annum, 10s. 6d. Foreign:—Per Annum, 15s. 2d.; payable, in all cases, in advance.

**NOTICE TO CORRESPONDENTS.**

All MS., Letters, Books for Review, and other matters intended for the Editor should be addressed The Editor, The Lodge, Porchester Square, London, W.

The Editor cannot undertake to return rejected MS., even when accompanied by stamped directed envelope.

**"Burdett's Hospital Annual," 1895.**

Sixth year of publication. 950 pp. Scarlet cloth, gilt lettered. Price 5s. A most complete guide to British, Colonial, Indian, and American Hospitals, Dispensaries, Nursing and Convalescent Institutions, and Asylums. A few complete sets of the preceding five issues of the "Annual" may still be obtained on application to the Publishers, The Scientific Press (Limited), 428, Strand, London, W.C.

**NOTICE.—Vol. XVIII. of "The Hospital" (April to September, 1895), in handsome cloth binding, is now on sale at the Office of this Journal. Price 7s. 6d. Cases for binding the Numbers contained in this Volume, 1s. 6d. Index to Vol. XVIII., 2½d., post free.**

**The Monthly Part for November is now on sale, price 6d.; by post, 8d.**

### SCRAPS AND GLEANINGS.

A Pathological and Bacteriological Institute is shortly to be provided for Dublin.

The workmen of Bunhope Colliery have contributed £54 to the Newcastle Hospital Fund.

An inaugural meeting of the Midland Medical Society was held last week at Birmingham.

A fire occurred at the Sheffield Infirmary recently, which, however was speedily extinguished, and no great damage was done.

The finances of the Northampton Infirmary are in a good condition; over £600 is the ordinary balance in hand from last year.

There were no fewer than 13,857 insane persons under official cognizance in Scotland in the early part of the present year.

The Duke of Richmond presided on Monday at a banquet at the Star and Garter Hotel, Richmond, in aid of the building fund of the Richmond Royal Hospital.

It is suggested that cycling sports shall be organised in the spring to augment the funds of the Swansea Hospital, which institution is again somewhat seriously involved financially.

The meeting at the Fulham Vestry Hall, on behalf of the so-called "Jubilee Hospital," was notable for the small attendance and the inconsequence of the remarks made by the majority of the speakers.

It is now arranged that the monument to be erected to perpetuate the memory of M. Pasteur at Paris is to assume an international character, and will be raised in co-operation with foreign subscriptions.

A temporary hospital has had to be improvised at St. Petersburg to meet the demands of an unusual prevalence of zymotic diseases in the Russian capital. At the present time there are actually 3,258 such cases to provide for, and the accommodation for the same is limited to 2,570.

The annual meeting of the Brackley Cottage Hospital (Banbury) has just taken place. An important addition to the hospital has been made by the purchase of a small garden at the rear of the building. Mr. Middleton has sent a cheque of 15 guineas to clear off certain liabilities of the hospital, which is doing a good work.

The eighty-sixth annual report of the Derbyshire Royal Infirmary shows an increase in the number of in-patients of 262, and out-patients 1,270 as compared with the previous year. The subscription list has also increased to an extent of £311. The past year has been one of considerable progress in the history of the institution.

It is being daily forced upon the inhabitants of Leyton that a hospital is very seriously needed; in fact, for a district with a population of 80,000, the accommodation at present existing is only for 16 patients. Just now, consequent upon an outbreak of scarlet fever and diphtheria, temporary accommodation by means of iron buildings is being provided.

A deputation of the Rhyl Urban District Committee have laid before the Duke of Westminster a request that the Hospital shall be erected in Marine Drive, instead of retaining its present site on the promenade. And to further facilitate this change the council have offered £2,600 and the owners of the adjoining property a further £800. The matter is yet under consideration.

The Bridgwater Infirmary has just had its annual meeting. The financial statement of this institution is of a highly satisfactory character, showing that the balance to the credit of the general fund has been increased from £68 at the beginning of the year to £241, and also a credit balance of £147 on the special fund for providing additional accommodation for the staff. An appeal is shortly to be made to the public for carrying out these very necessary alterations as soon as possible.

### THE STUDENT OF MEDICINE.

### APPOINTMENTS.

J. Brown, M.D.Vict., D.S.Sci., reappointed Medical Officer of Health for the Borough of Bacup and Physician to the Sourhall Fever Hospital. W. E. S. Burnett, L.R.C.P., L.R.C.S.Edin., appointed Medical Officer of Health to the Tintwistle Rural Sanitary District. W. McEntire Clendinnen, M.R.C.S., L.R.C.P. Lond., appointed Medical Officer of Health to the Coseley Urban District. Mr. T. Cooke, appointed Medical Officer of Health to the Limehurst Rural District Council. H. H. Dummere, L.R.C.P.Lond., M.R.C.S.Eng., reappointed Medical Officer for the No. 4 District of the Bromley Union. N. F. Edwards, M.B., Ch.B.Vict., appointed Assistant House Surgeon to the Swansea Hospital. R. M. Fenn, M.B., C.M., appointed Honorary Assistant Physician to the Manchester Hospital for Consumption and Diseases of the Throat and Chest. P. Fraser, M.D., B.Sc., appointed Medical Officer of Health to the Carnarvon Joint Sanitary Authority. D. Gibson, M.R.C.S.Eng., L.S.A., reappointed Medical Officer for the North Mynton District of the Hull Union. Dr. A. Hall, appointed Medical Officer of Health to the Mayfield Rural District. J. J. H. Jackman, L.R.C.P., L.R.C.S.I., appointed Medical Officer for Waterford Workhouse and Fever Hospital. P. James, F.R.C.S., appointed Surgeon to the Wellington Hospital, New Zealand. L. W. A. Keiffenheim-Trurridge, M.D.Durh., M.R.C.S., L.R.C.P., appointed Medical Officer of Health to the Hoo Rural Sanitary District. E. M. Knapp, L.R.C.P.Edin., M.R.C.S.Eng., appointed Medical Officer for the Aston, Ingham, and Linton Districts of the Ross Union. C. E. M. Lewis, M.A., M.B., B.C.Camb., appointed Assistant Resident Medical Officer to the North-West London Hospital. H. Littlejohn, M.B., D.P.H., C.M., appointed Medical Officer of Health for Scarborough. J. MacNidder, M.B., C.M.Edin., appointed Medical Officer for the Town District of the Hull Union. Dr. H. Morris, appointed Medical Officer for the Silverdale District of the Tunstall Union. T. H. P. Morris, M.R.C.S., L.R.C.P. Lond., appointed House Surgeon to the Stourbridge Dispensary. F. Oliphant, M.B.Edin., appointed Senior House Surgeon to the Chesterfield and North Derbyshire Hospital, Chesterfield. E. V. Phillips, L.R.C.P.Lond., M.R.C.S., appointed Medical Officer of Health to the Oxenden Rural Sanitary District. Dr. Powell, appointed Medical Officer of Health to the Llandyssul Rural District Council. R. Richmond, M.D., appointed Medical Officer of Health to the Dunmow Rural Sanitary District. Dr. Tibbitts, appointed Medical Officer of Health to the Quarry Bank Urban District Council. W. Trethowan, M.B., C.M.Aberd., appointed Honorary Assistant Surgeon to the General Hospital, Perth, West Australia. A. G. Tribe, L.S.A., appointed Surgeon of the Abergorky and Yniswen Collieries, Rhondda Valley. J. I. Twohig, L.R.C.P., L.R.C.S.Edin., appointed Medical Officer for the Goleen Dispensary District. O. M. White, M.R.C.S. Eng., L.S.A., reappointed Medical Officer for the No. 6 District of the Bromley Union. A. Wilson, L.R.C.P., M.R.C.S.Eng., reappointed Medical Officer for the South Mynton District of the Hull Union. A. E. Woodcock, L.R.C.P. and S.E., appointed Certifying Factory Surgeon. G. B. Wray, M.R.C.S. Eng., L.S.S., D.P.H., appointed Medical Officer of Health to the Basford District Council.

### VACANCIES.

The following vacancies are announced this week, the amount of salary in each case being given after the name of the institution:—

### Resident Medical Officers.

Denbighshire Infirmary, Denbigh.—House Surgeon; must be duly qualified to practise medicine and surgery, and be conversant with the Welsh language. Salary £80 per annum, with board, residence, and washing. Applications and testimonials to W. Vaughan Jones, Secretary, by December 2nd.

General Hospital, Birmingham.—Assistant House Surgeon. Appointment for six months. No salary, but residence, board, and washing provided. Applications to Howard J. Collins, House Governor, by November 30th.

New Hospital for Women, 144, Euston Road.—Two Qualified Medical Women as House Surgeons. Applications to Margaret M. Bagster, Secretary, by November 27th.

Tiverton Infirmary and Dispensary.—House Surgeon and Dispenser; registered and unmarried. Salary £105 per annum, with lodgings, attendance, fire, and lights. Applications to Arthur Fisher, Hon. Secretary, by November 25th.

London: THE SCIENTIFIC PRESS (LIMITED), 428, Strand, W.C.

### From A. and C. BLACK'S LIST.

**Text-Book of Operative Surgery.** By Dr. TH. KOCHER, Professor of Surgery and Director of the Surgical Clinic in the University of Bern. Translated by special permission of the Author from the Second Enlarged and Improved German Edition, by HAROLD J. STILES, M.B., C.M., Senior Demonstrator of Surgery in the University of Edinburgh; Assistant Surgeon, Royal Edinburgh Asylum for Sick Children. Illustrated with 185 cuts in text. Demy 8vo., cloth, price 20s.

**The Senile Heart.** Its Symptoms, Sequelæ, and Treatment. By GEO. W. BALFOUR, M.D., LL.D., Consulting Physician to the Royal Infirmary and to the Royal Public Dispensary, Edinburgh. Crown 8vo., cloth, price 5s.

**Plea for a Simpler Life.** By GEORGE S. KEITH, M.D., F.R.C.P.E. Crown 8vo., cloth, price 2s. 6d.

**Text-Book of General Pathology and Pathological Anatomy.** By Professor RICHARD THOMA, of Dorpat. Translated by ALEX. BRUCE, M.D., Lecturer on Pathology, Surgeons' Hall, Edinburgh. [*Vol. I. in preparation.*

A. AND C. BLACK, Soho Square, London, W.

### ROYAL NATIONAL PENSION FUND FOR NURSES.

*President*—**H.R.H. THE PRINCESS OF WALES.**

*Chairman*—WALTER H. BURNS, Esq. *Deputy-Chairman*—HENRY C. BURDETT, Esq.

PENSIONS SICKNESS. ACCIDENT.

### Total Invested Funds, £250,000.

Nurses are invited to join the Fund on account of the substantial and exceptional advantages which it offers them and which *they cannot obtain elsewhere.* The following are the chief points:—

1. **The Fund is Mutual and essentially Co-operative.**
2. **Easy Payment of Premiums.**
   Nurses can pay their premiums monthly or otherwise as best suits their convenience.
3. **The Fund is open to every Nurse.**
   Nurses can assure for Pensions of any amount commencing at any age.
4. **An Investment and Savings Bank.**
   Those entering under the returnable scale can have their premiums returned to them with compound interest, less a small deduction for working expenses. Interest allowed on deposits.
5. **Additions to Pensions.**
   Besides the Pension entered for, substantial additions thereto may be anticipated in the shape of bonuses.
6. **Sickness and Accident Assurance.**
   Policies are issued in connection with Pension policies assuring 10s., 15s., or 20s. a week in cases of incapacity from work through sickness or accident.

**Full particulars to be obtained from THE SECRETARY,**

ROYAL NATIONAL PENSION FUND FOR NURSES,

**28, FINSBURY PAVEMENT, LONDON, E.C.**

### "THE HOSPITAL"

AN INDEPENDENT JOURNAL OF

**The Medical Sciences AND Hospital Administration,**

WITH WHICH IS ISSUED WEEKLY

A SPECIAL

### SUPPLEMENT FOR NURSES.

PUBLISHED EVERY SATURDAY.

PRICE TWOPENCE.

Matrons, Sisters, or Nurses will find in The Hospital a chronicle of the latest Nursing News, Practical Articles of the highest value to those who wish to keep themselves abreast with the times, and brightly written articles from Nurses all over the world.

SUBSCRIPTIONS CAN COMMENCE AT ANY TIME.

### TERMS OF SUBSCRIPTION.

### WEEKLY ISSUE.

| | Great Britain. | Foreign and Colonial. |
|---|---|---|
| For One Year ... ... | 10s. 6d. ... | 15s. 2d. |
| For Six Months ... | 5s. 3d. ... | 7s. 7d. |
| For Three Months... | 2s. 8d. ... | 3s. 10d. |

### MONTHLY PARTS.

| | Post Free to any Part of the World. |
|---|---|
| For One Year ... ... | 8s. 6d. |
| For Six Months ... ... | 4s. 3d. |
| For Three Months ... ... | 2s. 2d. |

Postal Orders and Cheques should be made payable to "The Scientific Press, Limited."

N.B.—In order to prevent delay, all business communications should be addressed to "The Manager" only, and not to any person individually.

### CHANGE OF ADDRESS.

In event of change of address, notice should be forwarded to the Publishers by the Saturday previous to date of publication, and the old address should also be given.

"The Hospital" can be obtained of all Booksellers and Newsagents in the United Kingdom, and at all the Railway Stalls, of Messrs. W. H. Smith and Son; and also from the following Agents in the Colonies and Abroad:—

Paris: Librairie Galignani, 224, Rue de Rivoli.
Berlin: Messrs. A. Asher and Co.
Leipsig: Mr. F. A. Brockhaus.
New York: The International News Co.
Montreal: The Montreal News Co.
Toronto: The Toronto News Co.
Melbourne, Sydney, Adelaide, and New Zealand: Messrs. Gordon and Gotch.
India: At all the Railway Bookstalls and Agencies of Messrs. A. H. Wheeler and Co.
South Africa: Messrs. Juta, Cape Town.

PUBLISHING OFFICES: 428, STRAND, LONDON, W.C.

*In the Press. Price 5s.*

### BURDETT'S OFFICIAL NURSING DIRECTORY

A DIRECTORY, NOT A REGISTER.

THE HOSPITAL states: "It is well to bear in mind the difference and distinction which exists between a register and a directory. A register implies rights, and suggests that those who are not upon it are in some way or another inferior beings. But a directory is another matter. A directory gives no rights; insertion in it implies no adherence to any particular party or any particular opinion, and in no way interferes with entry on the register of any association, or the list of any institute, to which the individual may belong. All that a directory professes to do is to give information. The 'Medical Register' is not a mere list of qualified medical men; it is admission to the Register, which itself makes him qualified. For years, perhaps even now, 'Churchill's Medical Directory' contained the names of medical men who, so far as public appointments were concerned, were unqualified; it also contains the names of homœopathists, whom four out of every five medical men will not meet; and it certainly contains the names of many for whose moral character we should not like to vouch. But it gives full and accurate information about them all, and for the sake of that information it is constantly consulted both by the public and the doctors."

WHAT NURSES NEED.

"At present there is no Register for the nursing profession giving any legal status. Such a thing may come or it may not; but in either case there is no reason why there should not be a Directory, giving such information about the career of each nurse as both the public and the profession want; information, by the by, which no legal Register would be likely to insert. The 'Medical Register' is a bald and meagre thing compared with the 'Medical Directory.' What Burdett's 'Official Nursing Directory' proposes to do is to supply the want of a directory giving information. It will give no rights, it will imply no partisanship, it will give no guarantee except that what it prints is true, and is derived from official sources."

ACCEPTABLE TO ALL NURSES.

"This ought to be enough to make it acceptable to the public, and every nurse ought to make sure that, whatever list or register she may be on, her name shall also appear on Burdett's 'Official Directory.'" Applications for forms should be made at once to the Editor, "Burdett's Official Nursing Directory," 428, Strand, W.C.

**The above work may be subscribed for at a reduced price by those whose names appear in the book, and who supply full particulars on a form which can be obtained upon application to the Publishers,**

THE SCIENTIFIC PRESS (Lim.), 428, Strand, London, W.C.

### IMPORTANT NOTICE.

### OUR ISSUE OF DECEMBER 14TH

WILL CONSIST OF A

### SPECIAL DOUBLE CHRISTMAS NUMBER,

With which will be presented a handsome presentation plate of

**H.R.H. THE PRINCE OF WALES.**

Taken from an entirely new and hitherto unpublished photograph.

This plate will be a companion plate to that of H.R.H. the Princess of Wales, given with our Christmas Number in December last.

**2d.** PRICE AS USUAL. **2d.**

In order to secure copies orders should be sent in at once to

THE MANAGER, "THE HOSPITAL," 428, STRAND, LONDON, W.C.

### NURSING BOOKS AT FULL DISCOUNT.

*LIST POST FREE.*

The Theory and Practice of Nursing (Percy Lewis, M.D.), 3/6 for **2/8.**
The Nurse's Dictionary (Honnor Morten), 2/- for **1/6.**
The Nurse's Case Book, 6d. for **3½d.**

### STANDARD TEXT-BOOKS ON NURSING, &c.

READY NOVEMBER 25.

*8th Edition. Greatly enlarged and thoroughly revised. Crown 8vo., cloth gilt, 3s. 6d.*

### NURSING: ITS THEORY AND PRACTICE,

BEING A

COMPLETE TEXT-BOOK OF MEDICAL, SURGICAL, AND MONTHLY NURSING.

By **PERCY G. LEWIS, M.D., M.R.C.S., L.S.A., &c., &c.**

To this edition have been added three chapters on Monthly Nursing, together with additions to the chapters on Blood Diseases, on Antiseptics, on the Nursing of Children, and on Private Nursing. Under these headings will be found a short account of the new Aseptic Treatment, the Antitoxin and Serum Treatments, and the Treatment by Thyroid Extracts.

These additions, combined with the revision throughout of the text, render this work the best and most complete Text-book on Nursing in the market, and strengthen its position as a Classic in the Training Schools of the World.

Now Ready. With numerous Illustrations, demy 8vo., blue buckram, gilt, 3s. 6d.

### DIET IN SICKNESS AND IN HEALTH.

By **Mrs. ERNEST HART,**

*Formerly Student of the Faculty of Medicine of Paris, and of the London School of Medicine for Women.*

With an Introduction by Sir **HENRY THOMPSON, F.R.C.S., M.B. Lond.**

"Does not ask the reader blandly to accept such and such a diet for such and such complaint, but explains to him, briefly and simply, the why and wherefore, and gives in each case excellent physiological lessons, illustrated with comprehensive diagrams. Another good point is its cheerful sensible tone. The book is excellent in every way, and may be warmly recommended to the enormous number of people who, for one reason or another, have to be careful about their diet."—*Westminster Gazette.*

"Mrs. Hart not only expounds the principles of dietetics with insight and authority, applying them particularly and generally to all forms of disease and disability, but she is also able to act as a guide to the actual practice of dietetics. A really useful handbook to all invalids, nurses, medical students, and their kind."—*Science Siftings.*

Second and Revised Edition. Demy 16mo., suitable for the apron pocket, handsomely bound in cloth, 140 pp. Price 2s.; in handsome leather, gilt, price 2s. 6d. net.

**THE NURSES' DICTIONARY OF MEDICAL TERMS AND NURSING TREATMENT.**—Compiled for the use of Nurses by HONNOR MORTEN. Containing descriptions of the principal Medical and Nursing Terms and Abbreviations, Instruments, Drugs, Diseases, Accidents, Treatments, Physiological Names, Operations, Foods, Appliances, &c., &c., encountered in the Ward or Sick-room.

200 pp., Crown 8vo., cloth, 3s. 6d. Profusely Illustrated with Original Drawings, with list of Instruments required, and a Glossary of Terms.

**OPHTHALMIC NURSING.** By SYDNEY STEPHENSON, M.B., F.R.C.S.E., Surgeon to the Ophthalmic School, Hanwell, W., &c., &c. This is a valuable contribution to Nursing literature on a subject hitherto never handled with special reference to Nursing. On account of its compact and clear arrangement, and its numerous illustrations, it is specially recommended to the use of the Nursing Profession.

Second Edition, with a New Chapter and several Coloured Plates Illustrative of Feeding Bottles used in ancient times, &c. Crown 8vo., imitation cloth, 5s. With 10 plates and many Illustrations in the Text, and Fac-simile Autograph Letters from the late Sir Andrew Clark, M. Pasteur, &c.

**INFANT FEEDING BY ARTIFICIAL MEANS**: A Scientific and Practical Treatise on the Dietetics of Infancy. By S. H. SADLER, Author of "Suggestions to Mothers," "Management of Children," "Education."

Crown 8vo., cloth gilt, Illustrated, price 5s.

**HELPS IN SICKNESS AND TO HEALTH**: Where to Go and What to Do. Being a Guide to Home Nursing and a Handbook to Health in the Habitation, the Nursery, the Schoolroom, and the Person, with a chapter on Pleasure and Health Resorts. By HENRY C. BURDETT, Author of "Hospitals and Asylums of the World," "Pay Hospitals of the World," "Cottage Hospitals, General, Fever, and Convalescent," &c., &c.

"It would be difficult to find one which should be more welcome in a household than this unpretending but most useful book."—*The Times.*

Important New Handbook of Midwifery. Handsomely bound in leather, 260 pp., profusely illustrated, price 6s.

**A PRACTICAL HAND-BOOK OF MIDWIFERY.** By FRANCIS W. N. HAULTAIN, M.D. A Practical Manual produced in a portable and convenient form for reference.

Third Edition, enlarged and partly re-written, crown 8vo., cloth gilt, about 200 pp., price 2s. 6d.

**HOSPITAL SISTERS AND THEIR DUTIES.** By EVA C. E. LUCKES, Matron to the London Hospital. The favourable reception accorded to the first two editions of this useful and interesting work, and the regular demand for copies of it, encourage the publishers to place this third edition, greatly improved and enlarged, before the Institutions. The experience and position of its author entitle it to authority, and every Matron, Sister, or Nurse who aspires to become a Sister should read the advice and information contained in its pages.

Crown 8vo., 500 pp., Second Edition, 7 Plates, 18 Illustrations, Charts, &c., price 7s. 6d. net.

**NURSING.** By ISABEL ADAMS HAMPTON.

"It is not too much to say that in no single volume yet published has the subject been so scientifically, carefully, and completely treated as it has been by Miss Hampton. . . . Whether as a teaching manual or as a book of reference, the thoroughly practical instruction which it conveys is sure to obtain a wide circulation."—*The Hospital.*

Crown 8vo., cloth gilt, containing many Plates and Illustrations, price 2s. 6d.

**SPINAL CURVATURE AND AWKWARD DEPORTMENT**: Their Causes and Prevention in Children. Dr. GEORGE MULLER, Professor of Medicine and Orthopædics, Berlin. English Edition, edited and adapted by RICHARD GREENE, F.R.C.P.

"An able essay upon the effect produced by exercise, attitude, and movement upon the growth and development of the body. He further gives directions which any intelligent parent or teacher could carry out with the help of very simple apparatus, showing how to avoid or, in early cases, to cure certain malformations which, in the majority of cases, arise either from the neglect of very simple hygienic rules or from carelessness."—*Daily Chronicle.*

LONDON: THE SCIENTIFIC PRESS (LIMITED), 428, STRAND, W.C.

